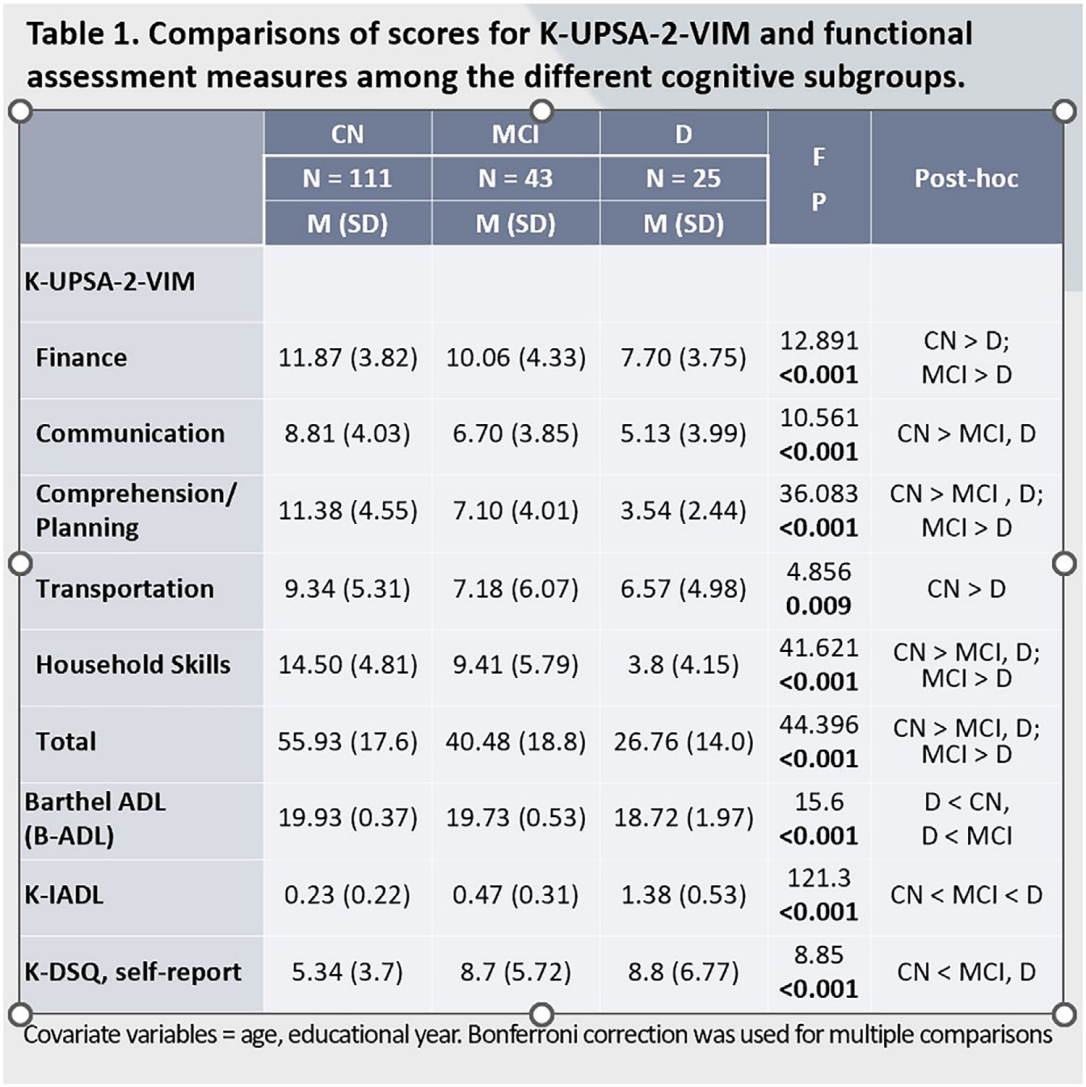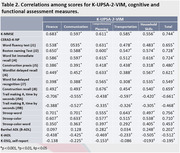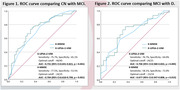# Validation of the measures for activities of daily living in older adult with cognitive disorders: the Korean version of the University of California San Diego Performance‐based Skills Assessment

**DOI:** 10.1002/alz.088050

**Published:** 2025-01-03

**Authors:** Seon Jin Yim, Kayoung Kim, Chaelin Joo, Joo Hyun Han

**Affiliations:** ^1^ National Center for Mental Health, Seoul Korea, Republic of (South)

## Abstract

**Background:**

The study aimed to evaluate the validity of the Korean version of the University of California San Diego Performance‐based Skills Assessment, Validation of Intermediate Measures (K‐UPSA‐2‐VIM) in patients with dementia (D), Mild cognitive impairment (MCI), cognitive normal control group (CN), and explore the usefulness of the instrument as a measure of ADL in older adults with cognitive disorder.

**Method:**

Study participants were 25 patients with D, 43 patients with MCI, 111 controls with CN group, respectively. **For cognitive assessment**, Mini Mental State Examination (K‐MMSE‐2), Consortium to Establish a Registry for Alzheimer’s Disease neuropsychological battery (CERAD‐K‐NP), Clinical Dementia Rating (CDR) were used. **For functional assessment,** Barthel‐Activities of Daily Living (B‐ADL), Instrumental Activities of Daily Living (K‐IADL), Dementia Screening questionnaire (K‐DSQ), K‐UPSA‐2‐VIM were used. Multivariate Analysis of Covariance (MANCOVA) was conducted to assess whether statistically significant differences existed in the scores of K‐UPSA‐2‐VIM and functional assessment measures among the different cognitive subgroups. Receiver Operating Characteristic (ROC) curve analyses were conducted to determine the optimal cut‐off scores for K‐UPSA‐2‐VIM that exhibit maximum sensitivity and specificity in discriminating between CN and MCI groups, MCI and D groups, in respectively.

**Result:**

Statistically significant differences were observed in all subdomains and total score of the K‐UPSA‐2‐VIM among three cognitive groups. All subdomains and the total score of K‐UPSA‐2‐VIM demonstrated a significant correlation with all subdomains of CERAD‐K‐NP. K‐UPSA‐2‐VIM demonstrated 75.7% of sensitivity and 65.1% of specificity, with an area under the curve (AUC) of 0.731 (95% CI:0.641‐0.821, p < 0.001) in discriminating between CN and MCI groups. In discriminating between MCI and D groups, 76.7% of sensitivity and 64.0% of specificity, with an AUC of 0.706 (95% CI:0.580‐0.833, p = 0.005) were demonstrated.

**Conclusion:**

Our study results suggest that the K‐UPSA‐2‐VIM has diagnostic validity in Korean patients with dementia and mild cognitive impairment. The K‐UPSA‐2‐VIM is useful for assessing patients, including elderly individuals living alone without caregivers.